# The Hierarchical Organization of the Layered Fibrous Shell of *Chamelea gallina* Guides Fracture Pathways

**DOI:** 10.3390/biom16091331

**Published:** 2026-09-13

**Authors:** Devis Montroni, Emilio Catelli, Silvia Prati, Arianna Mancuso, Stefano Goffredo, Giuseppe Falini

**Affiliations:** 1 Department of Chemistry “Giacomo Ciamician”, University of Bologna, Via Piero Gobetti 85, 40129 Bologna, Italy; 2 Department of Biological, Geological, and Environmental Sciences, University of Bologna, Via Selmi 3, 40126 Bologna, Italy; 3 Fano Marine Center, The Inter-Institute Center for Research on Marine Biodiversity, Resources and Biotechnologies, 61032 Fano, Italy

**Keywords:** aragonite, prismatic microstructure, crack deflection, biomineralization, *Chamelea gallina*

## Abstract

Molluscan shells combine mineralized layers with distinct microstructures that can influence crack propagation. Here, we provide a through-thickness, multiscale characterization of the all-aragonitic shell of the striped venus clam *Chamelea gallina* and relate its hierarchical textural organization to observed fracture-surface trajectories. Optical microscopy, scanning electron microscopy, energy-dispersive X-ray spectroscopy, and spatially resolved Fourier-transform infrared spectroscopy and X-ray diffraction resolve an outer region comprising (i) convergent and divergent fibrous sublayers separated by a preferred fracture plane, (ii) a porous transition layer with inverse-tulip microstructures, and (iii) a structurally distinct homogeneous inner layer whose crossed-lamellar organization becomes visible after etching. The key advance is the resolution of this outer architecture from nanogranule alignment to curved fibrous layers and a porous transition region, together with the identification of recurring changes in fracture-surface direction at its interfaces. FTIR and XRD reveal through-thickness variations in vibrational-band ratios and crystallographic parameters. As the fracture analysis is based on post-fracture morphology, crack steering and damage localization are discussed as plausible structure-related mechanisms supported by the observed features. The architecture suggests transferable design principles, including graded orientation, interface-guided deflection, and localized porosity, for mechanically robust bioinspired composites and provides a structural framework for future studies of shell evolution and function.

## 1. Introduction

Biological materials often derive multifunctional performance from hierarchical architectures spanning molecular to macroscopic length scales. Among them, biominerals are organic–inorganic composites in which organisms regulate composition, crystal organization, and interfaces to provide functions such as structural support, protection, predation, and food acquisition [1,2,3,4,5,6,7,8,9].

Molluscan shells are calcium carbonate-based biominerals containing a minor organic fraction, predominantly proteins and polysaccharides. Although this fraction is generally below 5 wt%, its distribution at crystal and layer interfaces, together with the hierarchical mineral architecture, contributes substantially to mechanical anisotropy, crack deflection, and other extrinsic toughening mechanisms [1,10,11,12,13,14,15,16,17,18,19,20,21].

Bivalve shells are among the most studied mineralized systems and are often described as comprising a nacreous inner layer and a prismatic outer layer. This arrangement is not universal: bivalves may combine nacreous, prismatic, crossed-lamellar, foliated, chalky, homogeneous, and other microstructures [1,3,22,23].

A common feature of these microstructures is hierarchical organization. Nacre commonly comprises stacked micrometric aragonite tablets separated by thin organic interlayers and is widely used as a model for crack deflection and interface-mediated toughening [11,12,24,25]. In crossed-lamellar shells, aragonitic lamellae are arranged in alternating orientations at successive hierarchical levels [23,26,27]. In several genera, including *Pinctada*, *Atrina*, and *Pinna*, the outer layer consists of elongated calcite prisms with polygonal cross-sections that grow approximately normal to the shell surface [28]. In oysters, the outer layer may instead contain porous chalky calcite or foliated calcite assembled from blade- or lath-shaped crystallites [29,30,31]. These architectures differ in porosity, crystallographic texture, and fracture pathways, demonstrating that mineral composition alone does not determine shell performance.

Although inner shell layers—especially nacre—have been studied extensively, the mechanical and structural roles of outer prismatic or foliated layers remain less well characterized. Nanoindentation of *Hyriopsis cumingii* showed higher hardness and elastic modulus in the prismatic layer than in the nacreous layer [32], while studies of *Crassostrea gigas* documented the strongly oriented foliated calcite architecture of the outer shell [29,31]. Together, these observations show that outer and inner layers can differ markedly in local mechanical response and crystallographic texture; their contribution to shell-scale fracture should therefore be evaluated as a function of mineral polymorph, texture, porosity, and loading mode.

*Chamelea gallina* (Linnaeus, 1758), the striped venus clam, is a commercially important venerid distributed in the Mediterranean and Black seas [33,34]. Previous studies have shown that its shell is entirely aragonitic and comprises structurally distinct outer and inner regions; they have also quantified environmental effects on shell geometry, porosity, fracture load, elastic response, strength, and fracture energy [33,35,36]. The specific hierarchical organization of the outer fibrous region and its relationship to observed fracture paths, however, remain incompletely resolved.

This study therefore characterizes the through-thickness morphology, nanogranular organization, composition, and crystallographic texture of the *C. gallina* shell, with particular emphasis on the outer fibrous region. We describe author-defined convergent, divergent, transition (“dissipation”), and homogeneous layers and evaluate how fracture surfaces intersect their internal interfaces. The objective is to identify candidate crack-steering features while explicitly distinguishing structural observations from mechanical properties that require direct testing.

## 2. Materials and Methods

### 2.1. Specimen Collection and Conservation

Commercially sized specimens of *C. gallina* (shell length over 22 mm) were collected off Cesenatico in the Adriatic Sea, using hydraulic dredges on subtidal soft bottoms at depths of 3–7 m. Following collection, the soft tissues were carefully removed from each specimen using a scalpel. The shells were cleaned with a toothbrush, rinsed with distilled water, and separated into right and left valves. Only right valves were analyzed in this study.

### 2.2. Sample Preparation

Samples were prepared according to the requirements of each analytical method. Shells were either split with a hammer to expose fresh fracture surfaces, ground to a powder in an agate mortar, or embedded in epoxy resin. Fractured surfaces were examined together with polished and etched sections so that fracture-specific topography could be distinguished from the underlying shell morphology.

For embedding, graded resin infiltration was used to promote the monomer penetration into the shell. The infiltration sequence comprised epoxy-resin mixtures in acetone of 50%, 75%, 90%, and 100% (*v*/*v*), with each step conducted for 30–60 min at room temperature. Samples were then immersed in fresh epoxy resin mixed with hardener, placed in silicone molds, and degassed repeatedly under vacuum to remove trapped air. The resin was cured overnight at room temperature.

The cured blocks were sectioned with a diamond saw and polished with alumina suspensions to a final particle size of 0.06 µm. Polished sections were sonicated in deionized water to remove residual surface contaminants before analysis.

The bleached sample that was used to observe the external surface of the shell was treated for 24 h with a 0.3–0.7% active chlorine NaClO solution (Merck KGaA, Darmstadt, Germany) with a 20 mg·mL^−1^ shell-to-solution ratio. After the treatment, the shell was soaked three times for at least 1 h in distilled water and then air-dried.

The etching of the polished section was performed treating a portion of the section with 20 µL of a 0.6–1.4% active chlorine NaClO solution (Merck) for 5 min followed by an abundant rinsing with distilled water, then treating the section area with 20 µL of an acetic acid solution (Merck) at pH 3.0 for 5 min and rinsing with distilled water, and finally repeating the treatment with NaClO for 2 min and rinsing with distilled water. The sample was then dried in a desiccator.

### 2.3. Optical Microscopy

**Figure 1:**
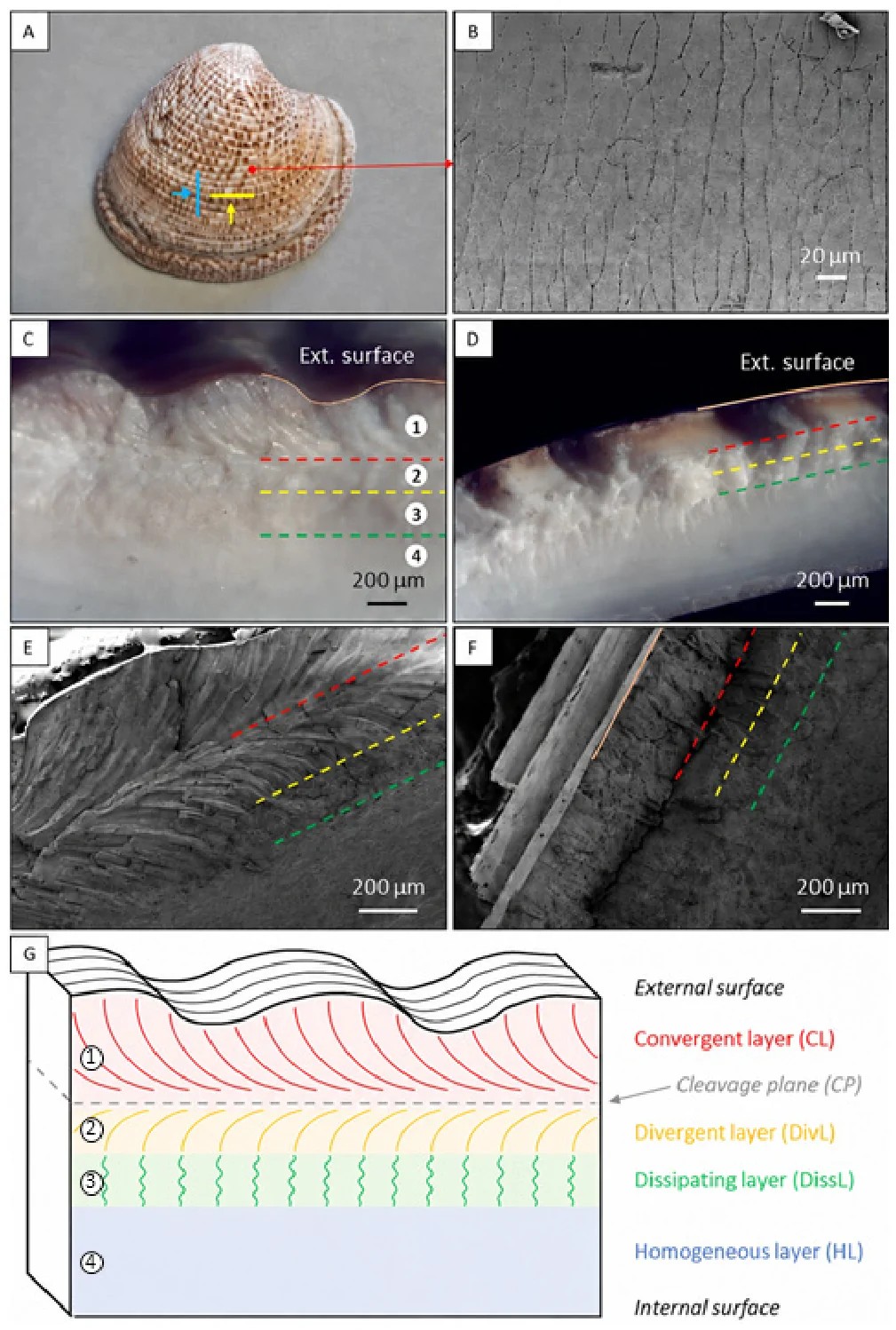
(**A**) Camera picture of a *Chamelea gallina* shell; the yellow arrow indicates the viewing direction for a transverse section, and the light-blue arrow indicates the viewing direction for a sagittal section. (**B**) SEM image of the bleached external shell surface, location identified by a red arrow in panel A. Optical micrographs (**C**,**D**) and SEM images (**E**,**F**) of sagittal (**C**,**E**) and transverse (**D**,**F**) sections. Dashed lines in (**C**–**F**) delimit the layers of the outer region. (**G**) Schematic representation of the overall organization in transverse section. The numbers indicate the different texture regions.

Optical micrographs reported in Figure 1 were acquired from shell cross-sections exposed by fracture. Images were recorded with a Leica L2 microscope (Leica Microsystems GmbH, Wetzlar, Germany) equipped with a 5.0 MP digital camera (Moticam 5+; Motic, Barcelona, Spain).

Optical microscopy observations associated with FTIR resultswere carried out using an Olympus BX51M optical microscope equipped with a digital scanner camera, Olympus DP70 (Olympus Corporation, Tokyo, Japan). The instrument has a fixed ocular of 10× and objectives with magnifications of 5×, 10×, 20×, 50× and 100×. For the present study, the images were acquired using the 5× and 10× objectives. Dark-field images were collected under illumination provided by a 100 W halogen projection lamp, whereas UV images were acquired using an Ushio Electric USH102D ultraviolet lamp (Olympus Corporation, Tokyo, Japan).

### 2.4. Scanning Electron Microscopy (SEM) and Energy-Dispersive X-Ray Spectroscopy (EDS)

SEM was performed on freshly fractured shell fragments. Each fragment was mounted on carbon adhesive tape with the selected cross-section exposed and was coated with a nominal 10–20 nm layer of gold before imaging. Secondary-electron images were acquired with a Zeiss LEO 1530 field-emission SEM (Carl Zeiss, Oberkochen, Germany) at an accelerating voltage of 5 kV, a 20 µm aperture, and a working distance of 9 mm.

EDS measurements were performed on polished, resin-embedded shell sections with a Zeiss EVO LS10 environmental SEM (Carl Zeiss, Oberkochen, Germany) equipped with a LaB_6_ thermionic source, an Oxford Instruments energy-dispersive X-ray system with a silicon-drift detector (Oxford Instruments, Abingdon, UK), and Quantax System software Esprit 2.0. Spectra were acquired at 15 kV, 276 pA and a working distance of 8.5 mm, with an acquisition live time of approximately 10 min.

### 2.5. Fourier-Transform Infrared Spectroscopy (FTIR)

Attenuated total reflectance (ATR)-FTIR spectra of dry shell powders were acquired with a Thermo Scientific Nicolet iS10 spectrometer (Thermo Fisher Scientific, Waltham, MA, USA) fitted with a germanium crystal and processed in OMNIC 9.8.286 software (Thermo Electron Corp., Woburn, MA, USA). Each spectrum was collected at a spectral resolution of 2 cm^−1^ from 300 co-added scans.

FTIR mapping analysis was performed using a Thermo Scientific Nicolet iN™10MX imaging microscope (Thermo Fisher Scientific, Waltham, MA, USA), equipped with a liquid nitrogen-cooled mercury cadmium telluride detector. Measurements were carried out in Attenuated Total Reflection (ATR) mode using a slide-on conical germanium ATR crystal. Spectra were acquired in the range 4000–675 cm^−1^ spectral range with a spectral resolution of 4 cm^−1^. For the cross-section, the FTIR map was collected over an area of 80 × 2200 μm^2^, using a step of 20 μm in the x and y directions. An aperture size of 80 × 80 μm^2^ was employed, corresponding to an effective area of analysis of about 20 × 20 μm^2^ at each measurement point. The map was subsequently cropped to remove the edge regions containing signals from the embedding resin. Spectral processing, including water vapor and baseline correction, as well as the calculation of peak intensity ratios was performed using OMNIC Picta™ software v1.0 (ThermoFisher Scientific, Waltham, MA, USA).

### 2.6. X-Ray Diffraction (XRD)

X-ray diffraction patterns were collected with a Malvern Panalytical Empyrean diffractometer (Malvern Panalytical B.V., Almelo, The Netherlands) equipped with MultiCore optics (iCore and dCore) and a PIXcel3D detector. Cu K_α_ radiation (λ = 1.54056 Å) was generated at 40 kV and 40 mA; the goniometer radius was 240 mm.

Polished shell sections were scanned across the shell thickness at 100 µm intervals using an automated XY stage.

Patterns were recorded over 2θ = 24–50° with a step size of 0.026° and time per step of 400 s. To restrict the irradiated region, a 2 mm mask was used with a divergence-slit-to-sample distance of 95.50 mm, a 1/50° divergence slit, and a 1/32° antiscatter slit. This geometry yielded a nominal beam height of approximately 33 µm and a calculated irradiated area of 2.0 × 0.154 mm at 2θ = 25° and 2.0 × 0.079 mm at 2θ = 50°.

The powder pattern of the homogenized shell was collected on the same instrument using a spinning sample holder set to 2 s per revolution, a 5 mm mask, and 1/4° slits. The diffraction pattern was acquired using analogue acquisition parameters.

Peak positions and full widths at half maximum (FWHM, β) were obtained after profile fitting in X’Pert HighScore Plus (version 2.2d; Malvern Panalytical). Apparent coherent-domain sizes (D_hkl_) were estimated using the Scherrer equation:

$$D_{\mathrm{hkl}}=\frac{\left(K\;\times \;\lambda \right)}{\left(\beta \times cos\;\theta \right)}$$

where K = 0.9 is the shape factor, λ is the Cu K_α_ wavelength, β is the FWHM in radians, and θ is the Bragg angle.

## 3. Results

### 3.1. Morphology and Microstructure (SEM)

The shell of *C. gallina* (Figure 1A) displayed concentric growth lines externally and a smooth, pearly but non-iridescent inner surface. SEM examination of the bleached outer surface revealed elongated subdivisions, typically tens of micrometers wide and oriented predominantly perpendicular to the growth lines (Figure 1B).

Cross-sections revealed a multilayered architecture whose appearance depended on section orientation (Figure 1C–F). Here, “transverse” denotes a section tangential to a growth line, whereas “sagittal” denotes a section perpendicular to a growth line. Four morphologically distinct mineralized regions were resolved most clearly by SEM.

Polished sections additionally displayed a square-wave-like profile (revised Figure S1, which includes additional supporting SEM images).

**Figure 2:**
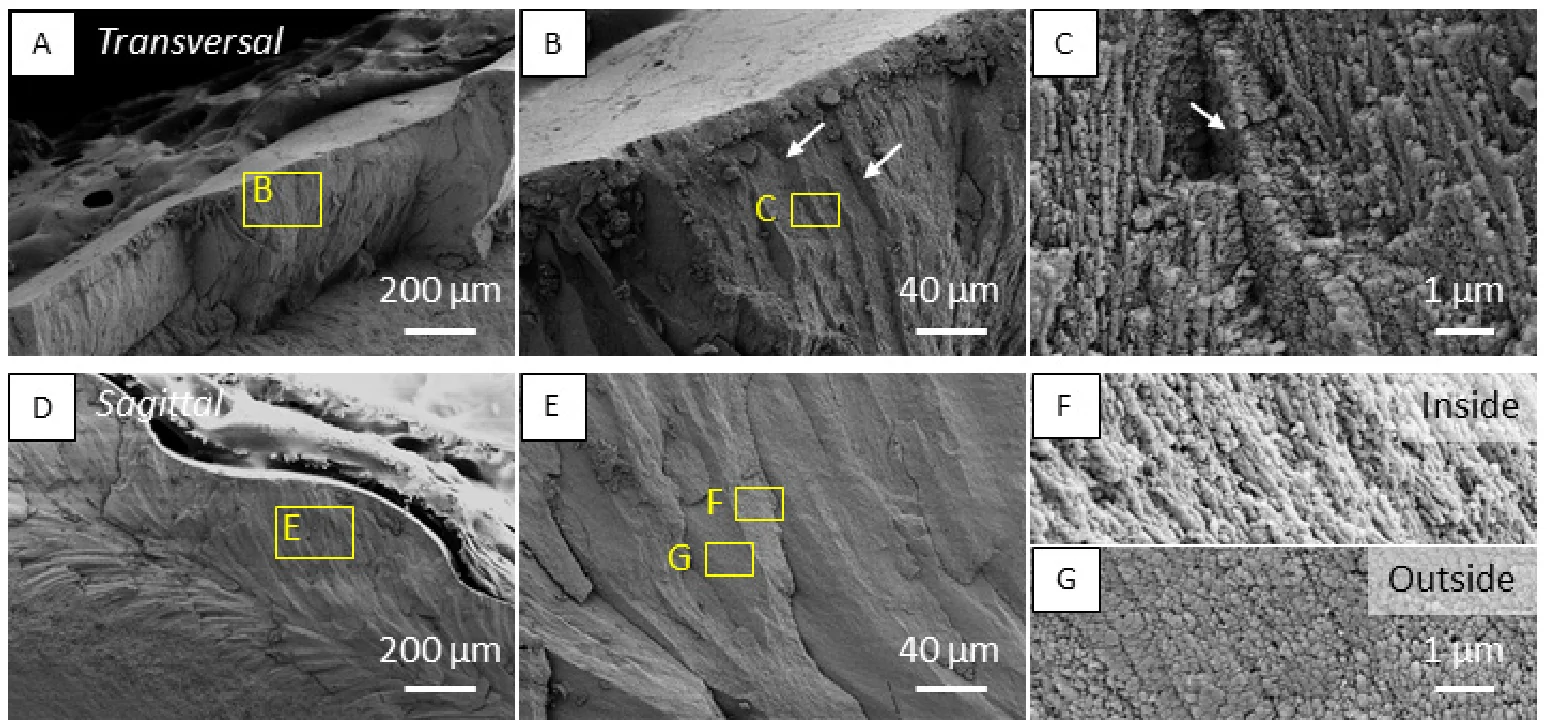
SEM images of the convergent layer (CL). (**A**–**C**) Transverse and (**D**–**G**) sagittal fracture surfaces shown at increasing magnification. White arrows in (**B**,**C**) mark the boundary between adjacent subdivisions. Panels (**F**,**G**) show the nanogranular organization inside a fiber and on its external fracture surface, respectively. Yellow squares within some of the panels identify the location in which the image in other panels where acquired.

A 10–20 µm thick, featureless surface layer was also visible (Figure 2B) and was tentatively assigned to the periostracum based on its position and appearance. Because the present study focuses on the mineralized architecture, this layer was not characterized further.

### 3.2. Convergent Layer

Immediately beneath the putative periostracum, the first mineralized region was designated as convergent layer (CL). In sagittal section, the CL exhibited a fibrous morphology where fibers were nearly normal to the external surface at their outer ends and progressively curved until they became approximately parallel to the interface with the underlying layer. At higher magnification, the fibers were composed of aligned, approximately spherical nanogranules, locally coalesced into short rod-like units (Figure 2F). The nanogranules were approximately 100 nm in diameter. At fiber–fiber boundaries, coalescence and alignment were less pronounced, and nanogranules from adjacent fibers formed a distinct interface (Figure 2C,G).

Fracture surfaces showed paths broadly aligned with the fiber direction and converging toward the boundary with the underlying layer. Separation occurred preferentially along fiber–fiber interfaces, and comparatively few fracture surfaces exposed the internal nanogranular structure of individual fibers. Thus, the recurring relationship at both hierarchical levels is interface-guided separation: fiber boundaries channel the observed fracture surface, while fiber curvature redirects it toward the CL–DivL boundary.

In transverse section, the subdivisions visible at the outer surface (Figure 1B) extended through the CL and formed distinct boundaries that were not crossed by the observed fibers. Near these boundaries, the nanogranular organization changed: elongated units were oriented approximately perpendicular to the subdivision boundary rather than parallel to the fiber axis (Figure 2C).

**Figure 3:**
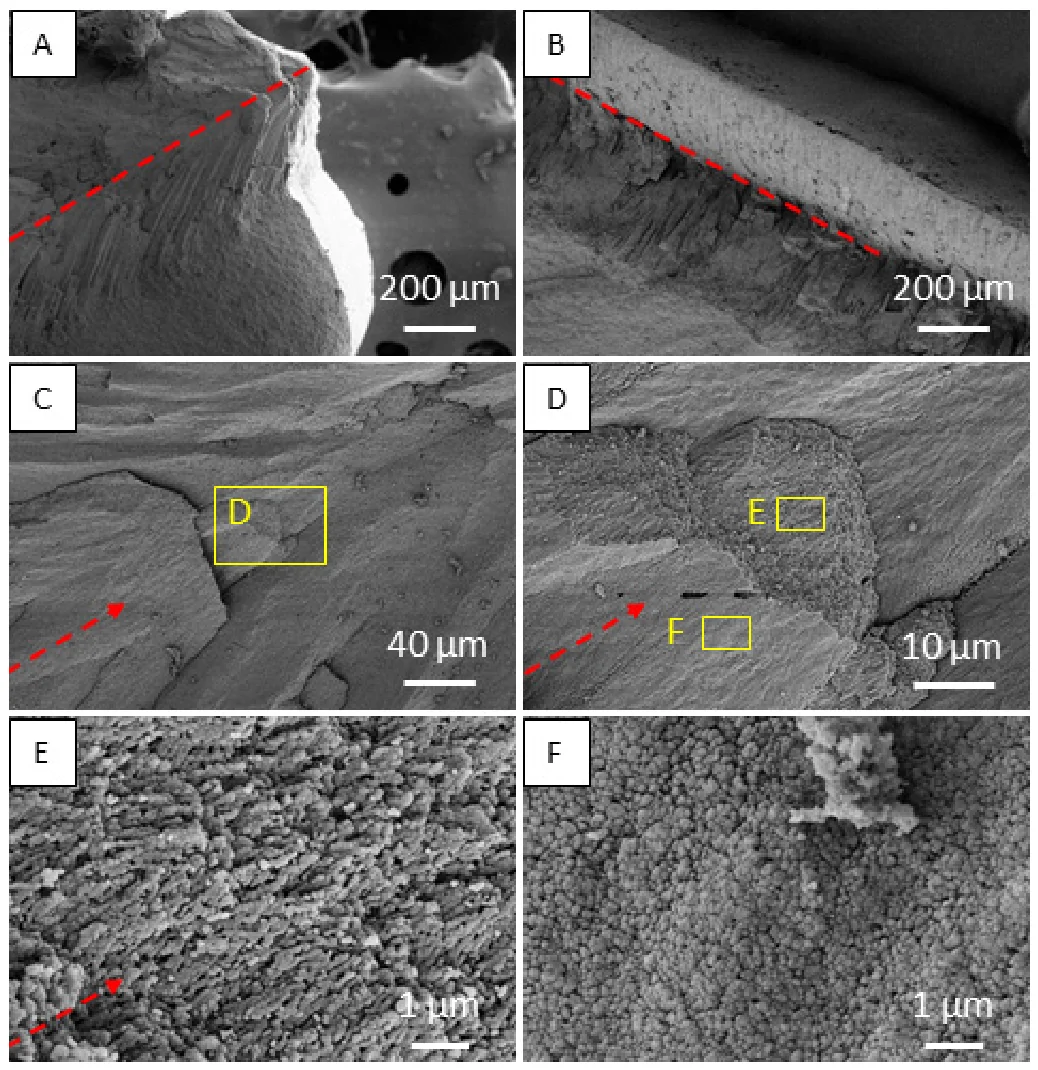
SEM images of the cleavage plane (CP) in (**A**) sagittal and (**B**) transverse sections, showing the V-shaped fracture morphology where the fracture surface intersects the CP. Magnified views in (**C**,**D**) show fibers approaching the CP from the CL and DivL. Panels (**E**,**F**) show the nanogranular organization inside a fiber and on its external fracture surface, respectively. The red dashed arrow/line in (**A**–**E**) indicates the CP orientation.

Fracture surfaces preferentially followed the boundaries between adjacent subdivisions. This anisotropy is consistent with the smoother and more regular fracture morphology observed in sagittal sections (Figure 1E, Figure 2D and Figure 3A).

### 3.3. Divergent Layer

The layer beneath the CL was designated the divergent layer (DivL). Its fibrous units curved in the opposite sense to those of the CL. The interface between the two layers coincided with a frequently exposed fracture plane, termed the cleavage plane (CP; Figure 3). Fracture surfaces crossing this boundary displayed an approximately V-shaped path (Figure 3A). Fibrous units and nanogranular alignments adjacent to the CP were oriented approximately parallel to the plane (Figure 3C–E).

**Figure 4:**
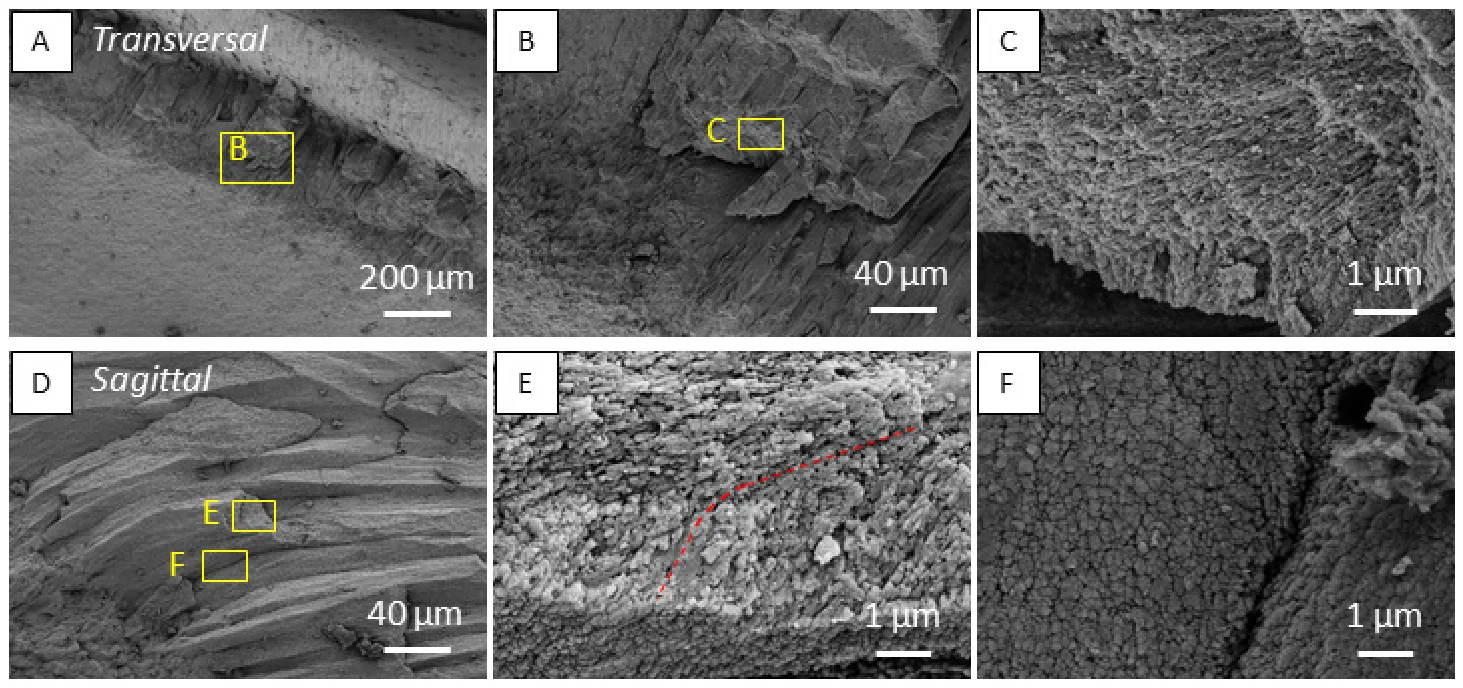
SEM images of the divergent layer (DivL) in (**A**–**C**) transverse and (**D**–**F**) sagittal sections. Panels (**C**) and (**E**) show the internal nanogranular organization of the fibers; red dashed lines indicate the change in nanogranule alignment near the fiber boundary. Panel (**F**) shows the nanogranular organization on an external fiber surface.

Compared with the CL, DivL fibers appeared thinner, shorter, and more sharply delineated. Their orientation changed from approximately parallel to the CP toward a more inwardly directed angle, reaching about 45° within the observed layer. At higher magnification, the intra-fiber nanogranules appeared more extensively coalesced and aligned than in the CL, whereas no obvious change in nanogranule size was resolved. Fiber–fiber interfaces remained distinct and were composed largely of facing, approximately spherical nanogranules (Figure 4F). Near these interfaces, nanogranule orientation progressively rotated from the fiber axis toward an orientation approximately normal to the boundary (Figure 4C,E).

In transverse section, the large subdivisions characteristic of the CL were no longer apparent, and a denser network of fiber–fiber interfaces was visible. Fracture surfaces preferentially followed these interfaces, whereas fractures intersecting a fiber tended to curve toward a neighboring fiber–fiber boundary.

### 3.4. Dissipation Layer

**Figure 5:**
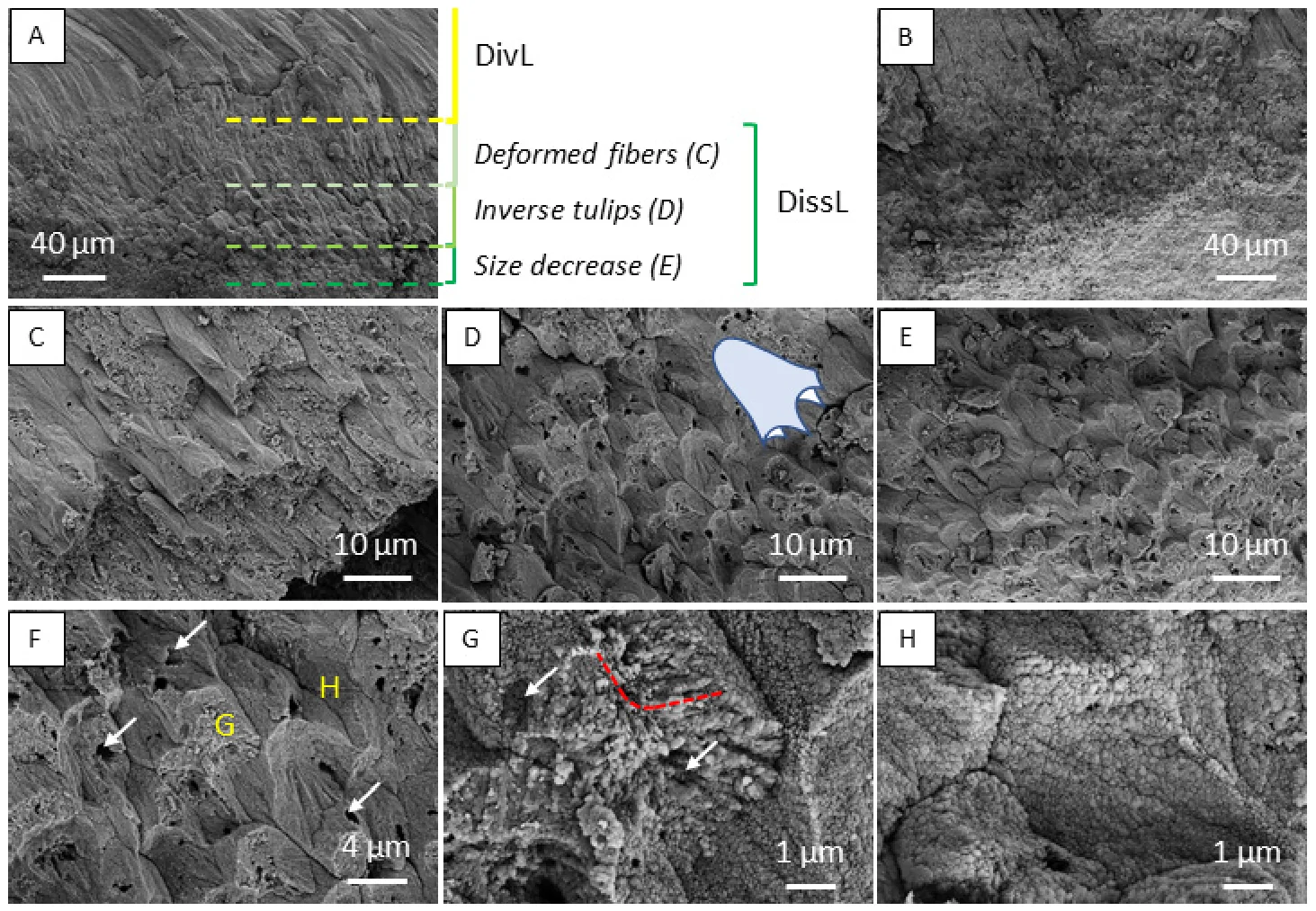
SEM images of the dissipation layer (DissL) in (**A**) sagittal and (**B**–**H**) transverse sections. Panel (**A**) summarizes the morphological sequence within this region and identifies for each layer the corresponding panel, and panels (**C**–**E**) show representative stages. Panel (**D**) includes a schematic of an inverse-tulip unit. White arrows in (**F**,**G**) mark pores and defects in the inverse-tulip region. Panels (**G**,**H**) show the nanogranular organization inside an inverse-tulip unit and on its external fracture surface, respectively. The red dashed line in (**G**) indicates the local nanogranule alignment.

Moving inward, the DivL gradually transitioned into the transition region between the outer layer and the internal layer of the shell; this porous transition layer has been designated as the dissipation layer (DissL). The term is retained here as a morphological label; direct energy-dissipation measurements were not performed. The region lost the continuous fibrous appearance of the outer layers but retained a nanogranular organization at higher magnification. No obvious change in nanogranule size was resolved. Across the transition between the DivL and the DissL, fibers became shorter and developed curved interfaces (Figure 5C). Over several tens of micrometers, they evolved into asymmetric units with a rounded cap and concave lateral margins. Because this outline resembles an inverted lily-flowered tulip, these units are referred to descriptively as “inverse-tulip” structures (Figure 5D). Numerous pores were visible in the same region (Figure 5F,G).

The interiors of the inverse-tulip units displayed a more pronounced version of the DivL alignment pattern. Nanogranules showed limited coalescence but strong local alignment: they were oriented along the long axis in the core and progressively diverged toward an orientation approximately normal to the external interface (Figure 5G).

The inverse-tulip units persisted for several tens of micrometers. Their size and aspect ratio then decreased progressively, the visible pores became less abundant, and the organization transitioned toward that of the underlying layer (the “size-decrease” region in Figure 5A,E).

Fracture surfaces in the DissL preferentially followed the interfaces between adjacent units. Where a fracture intersected a unit, the path commonly curved toward an interface, producing concave fracture surfaces.

### 3.5. Homogeneous Layer

**Figure 6:**
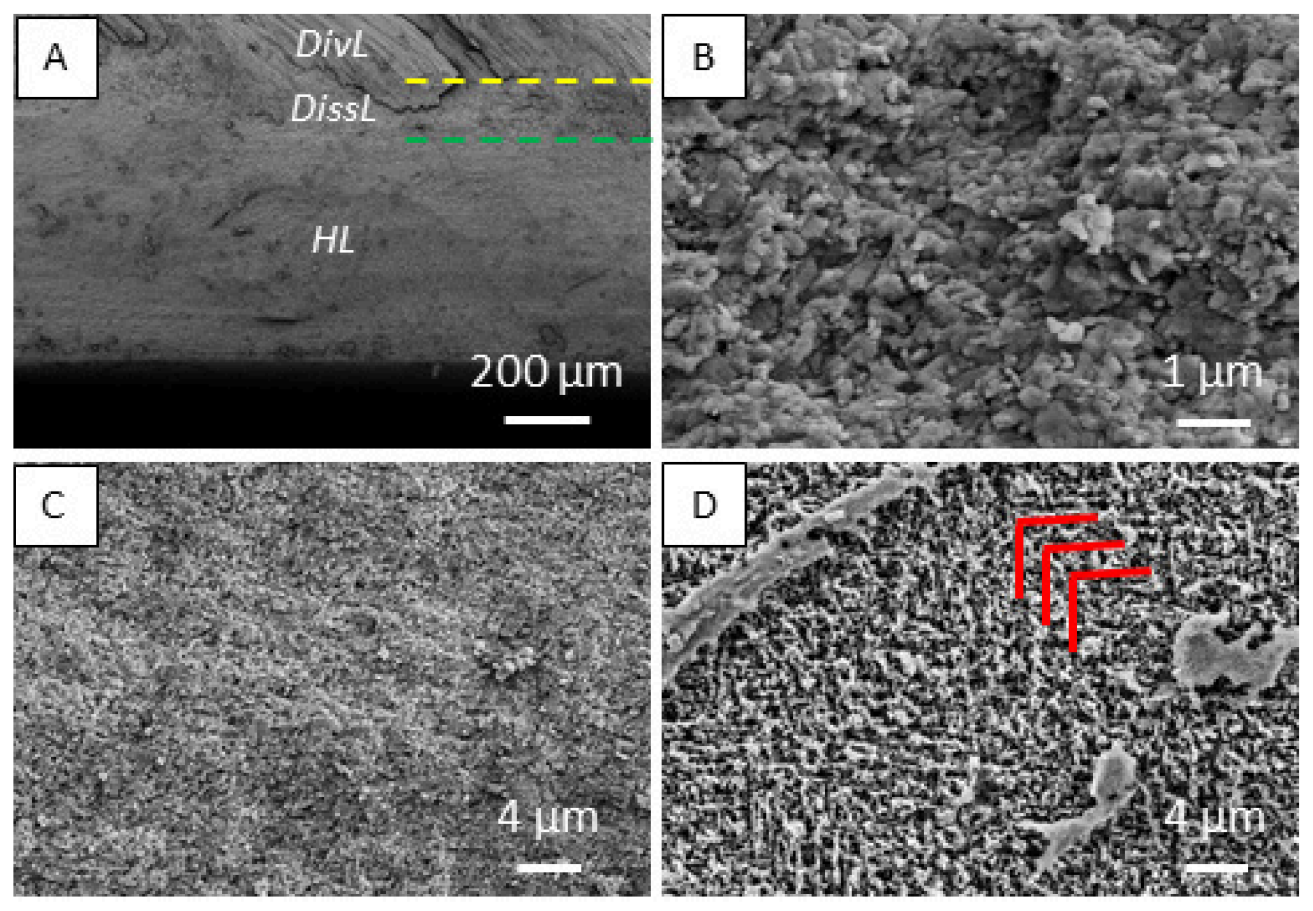
(**A**) SEM images of the homogeneous layer (HL) in sagittal section. (**B**) Higher-magnification image showing the densely packed nanogranular organization. Panels (**C**,**D**) show the HL before and after etching, respectively, revealing an underlying crossed-lamellar organization (highlighted with red lines).

Beneath the DissL, the innermost region appeared relatively featureless at low magnification and was therefore designated the homogeneous layer (HL; Figure 6A). At higher magnification, it consisted of densely packed, irregular nanogranules without any obvious preferred orientation in the fracture surfaces examined (Figure 6B).

After mild etching, an underlying crossed-lamellar organization became visible (Figure 6C,D), indicating that the term “homogeneous” refers only to its unetched appearance at the examined scale.

Fracture surfaces in the HL were predominantly conchoidal and tended to extend approximately parallel to the inner shell surface.

### 3.6. Structural and Compositional Characterization (XRD, FTIR, and EDS)

**Figure 7:**
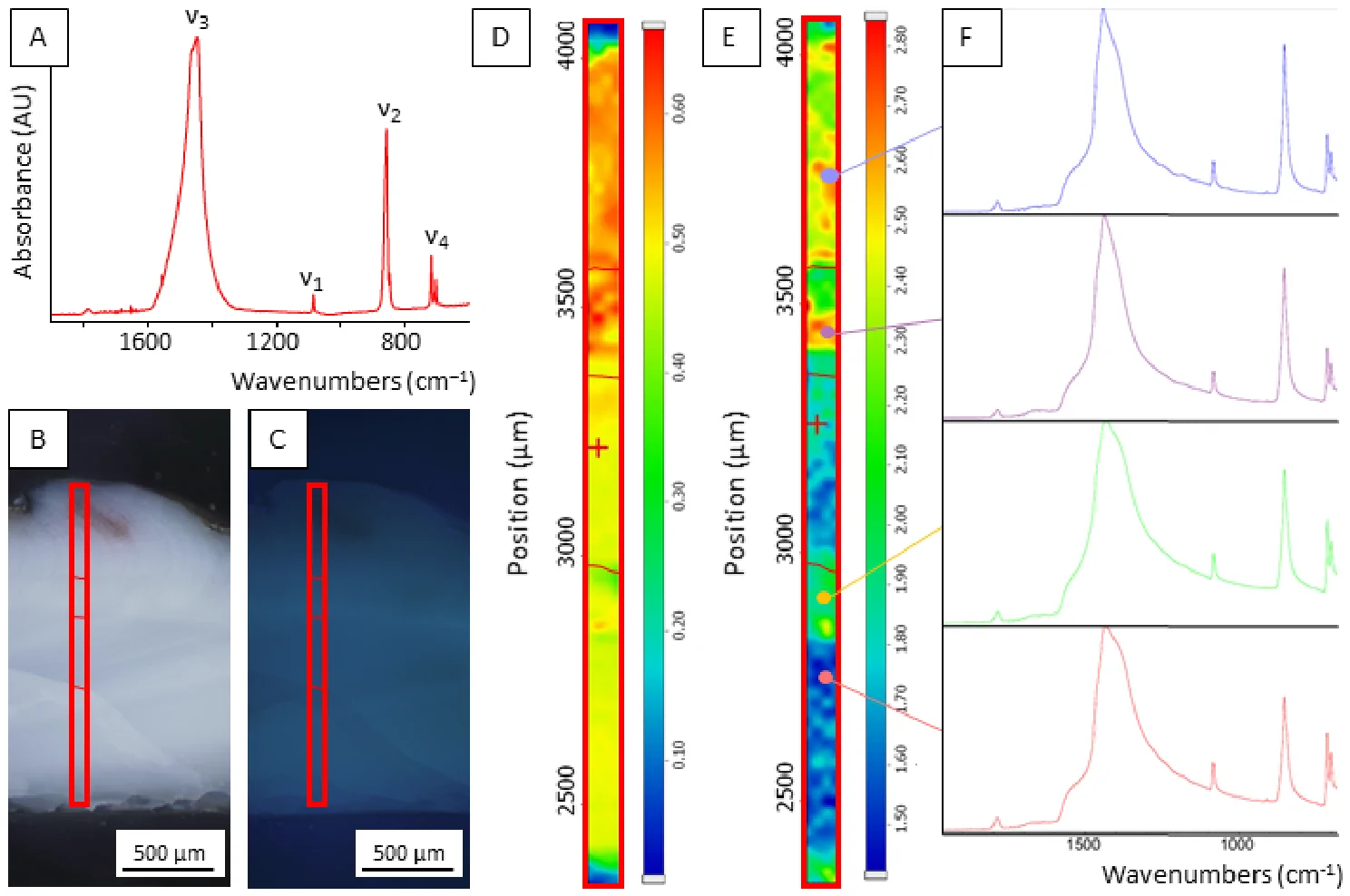
(**A**) ATR-FTIR spectrum of powdered shell. (**B**) Visible and (**C**) UV images of the investigated area (red rectangles). (**D**) FTIR map of the ν_3_-band intensity, (**E**) map of the ν_2_/ν_4_ intensity ratio across a sagittal shell section, and (**F**) spectra extracted from the different areas of the shell showing different heights of the ν_2_ and ν_4_ band intensities.

**Figure 8:**
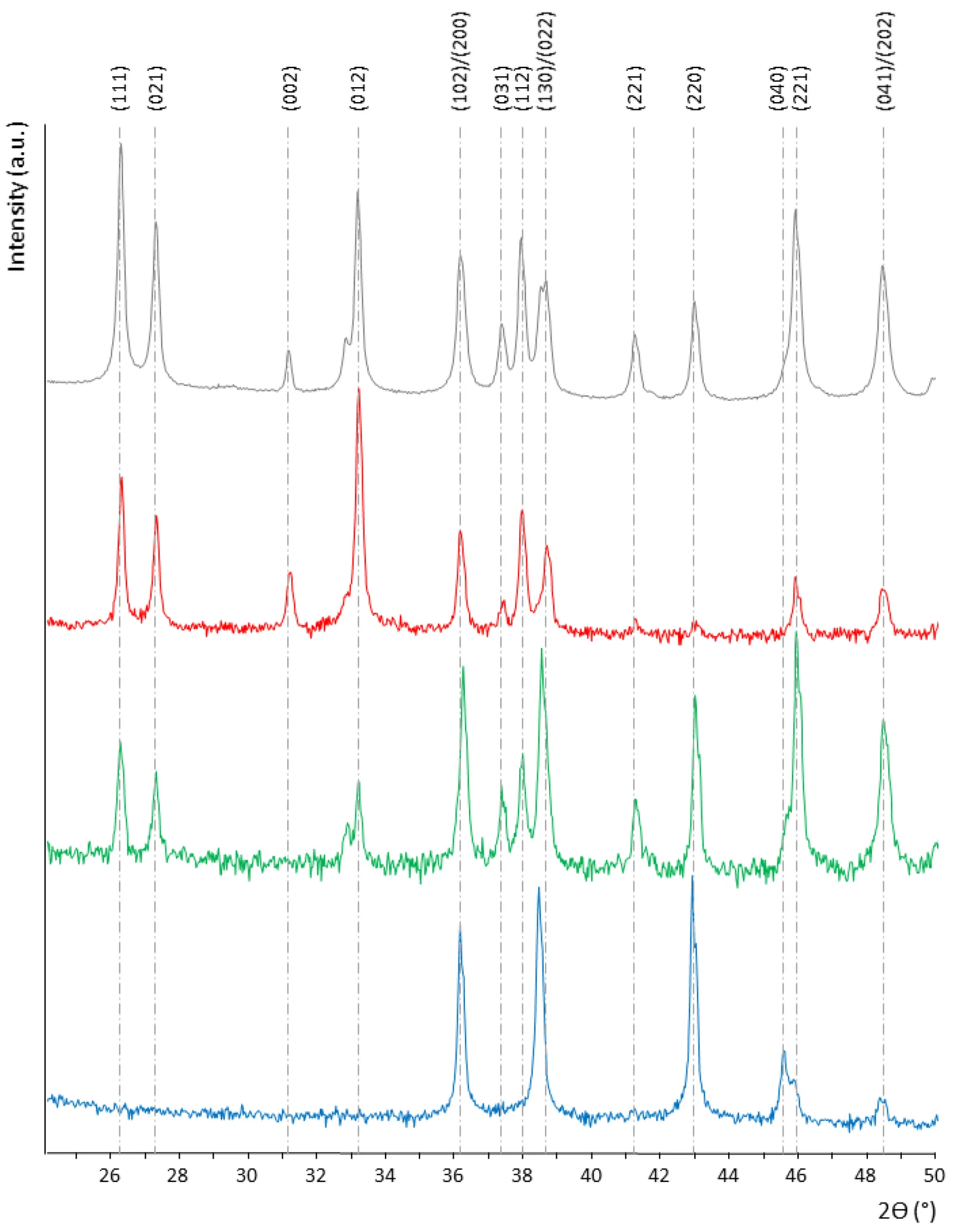
Normalized X-ray diffraction patterns of the *C. gallina* shell. The gray trace is the powder pattern of the homogenized shell; section patterns were collected from the transverse section near the external surface (red), in the middle of the shell (green), and near the internal surface (blue). Each pattern was normalized to its maximum intensity. Reflections are indexed to orthorhombic aragonite (Pmcn). Unresolved neighboring reflections are labeled jointly as (102)/(200), (130)/(022), and (041)/(202).

FTIR, EDS, and XRD were used to assess through-thickness structural and compositional variation. Powder FTIR and XRD identified aragonite as the only mineral phase resolved by these methods (Figure 7A and Figure 8). No organic bands were resolved under the acquisition conditions used. EDS detected minor Sr, Na, S, and Cl signals. Elemental maps and line scans did not resolve systematic layer-dependent compositional differences within the spatial resolution and detection limits of EDS (Figures S2 and S3).

The FTIR map was evaluated using the intensity ratio of the aragonite ν_2_ band near 860 cm^−1^ to the ν_4_ band near 713 cm^−1^ (Figure 7C). This ratio varied through the shell thickness and delineated three broad regions: an outer region with intermediate-to-high ratio values (coded in a green-to-red color scale), a middle region with higher values (yellow-to-red color scale), and a thicker inner region with lower values (coded in a blue color scale). In biomineral studies, Iν_2_/Iν_4_ has been used as an empirical index of structural disorder or amorphous calcium carbonate content, with higher values indicating a larger disordered or amorphous contribution rather than higher crystallinity [37,38,39,40,41]. In the present spatially resolved ATR measurements, however, relative band intensities may also be affected by crystallographic texture, local contact, and sampling geometry; accordingly, the ratio is interpreted only as a spatially varying spectral parameter.

The optical and SEM images of polished sections did not provide boundaries that could be registered unambiguously with the FTIR map. Consequently, the FTIR regions cannot be assigned with certainty to the SEM-defined layers. On positional grounds, the outer intermediate-ratio region may overlap the CL, whereas the inner low-ratio region may overlap the HL; any correspondence involving the DivL and DissL remains tentative.

XRD confirmed aragonite as the only crystalline phase detected in the powdered shell (Figure 8, gray pattern), and all labeled reflections were indexed to orthorhombic aragonite. The powder pattern displayed the broad set of reflections expected from a specimen with substantially reduced preferred orientation relative to the intact shell sections.

Spatially resolved XRD patterns were collected at 100 µm intervals across a shell section. Relative reflection intensities changed systematically from the outer to the inner surface, demonstrating strong through-thickness variation in preferred orientation along both the transverse (Figure S4) and the sagittal (Figure S5) section.

As with FTIR, associations between the XRD scan and the SEM-defined layer boundaries were limited by sample alignment and the absence of clear morphological markers on the polished section. Also, because the scans were normalized individually and acquired from a curved, polished cross-section with a finite beam footprint, these profiles provide qualitative evidence of texture changes rather than a quantitative orientation-distribution function.

Among the two directions examined, the transverse section was the one showing the most distinct changes across its cross-section. The intensity evolution can nevertheless be described in terms of three broad diffraction regimes (Figure 8).

The outermost pattern, tentatively associated with the CL, was dominated by the (012) reflection at 2θ ≈ 33.1°, with strong (111) and (021) reflections at approximately 26.2° and 27.2°, respectively. Contributions from (112), (102)/(200), (130)/(022), and (002) were also present. The number of enhanced reflections is consistent with a comparatively broad or multicomponent texture, although a one-dimensional θ–2θ scan cannot uniquely determine the orientation distribution.

Near the middle of the section, tentatively close to the inner boundary of the DissL, the relative intensity distribution changed markedly. Such a change was observed as a gradual shift between consecutive diffraction patterns. The unresolved (130)/(022) feature at approximately 38.4–38.6° became dominant, followed by the (220) reflection near 42.9° and the (102)/(200) feature near 36.1°. The (221) and (041)/(202) features were also prominent, whereas the low-angle (111), (021), and (012) reflections were strongly reduced relative to the outer region. This pattern defines a transitional texture rather than a uniformly attenuated version of the outer pattern.

Closer to the inner surface, the pattern gradually changed again and was tentatively assigned to the HL. Most of the intensity was concentrated in the (220), (130)/(022), and (102)/(200) features, whereas the low-angle reflections prominent in the CL were strongly suppressed. This selectivity indicates a different preferred orientation.

**Figure 9:**
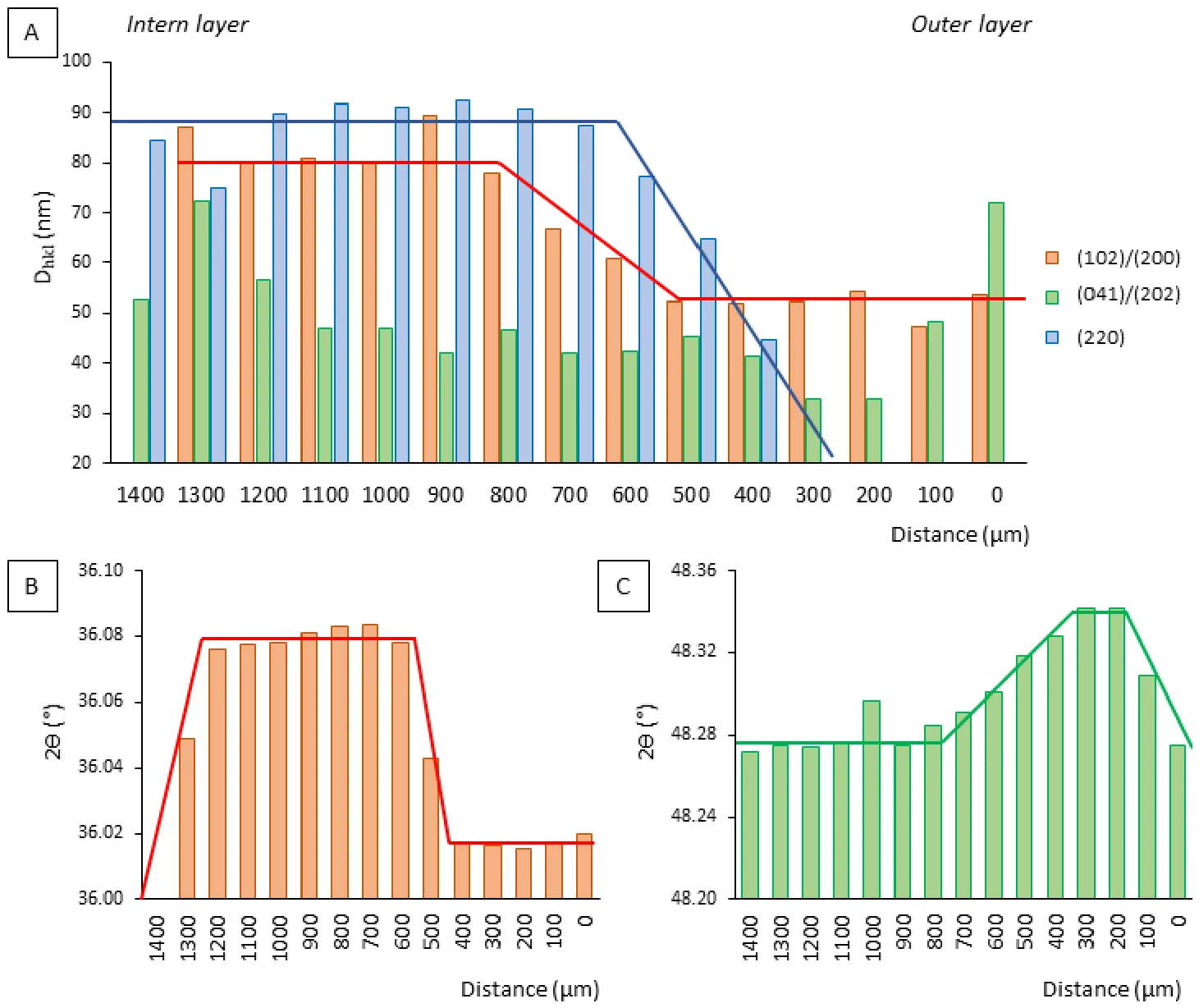
Analysis of the (200), considered prominent over (102), (041), considered prominent over (202), and (220) reflection contributions along the transverse section. (**A**) Apparent coherent-domain size as a function of position along the transverse cross-section; distance zero corresponds to the external surface. No (200) contribution could be fitted below 400 µm. Fitted peak positions of the (**B**) (102)/(200) and (**C**) (041)/(202) contributions are also shown. For each analyses a solid line identifies the results trend.

To examine the spatial trends in greater detail, the unresolved (102)/(200) and (041)/(202) features and the (220) reflection were fitted across the scan (Figure 9). Apparent coherent-domain sizes assigned to the (200), considered prominent over (102), and (220) contributions increased toward the inner half of the section, whereas values associated with the (041) contribution showed no clear systematic trend. Because two of the analyzed peaks contain overlapping reflections, reflection-specific Scherrer sizes depend on the validity of the deconvolution and should be interpreted cautiously.

Within the same area where the coherent-domain sizes changed, the fitted (200) peak shifted to higher 2θ values toward the HL, corresponding by Bragg’s law to a decrease in the associated d-spacing. A shift of the fitted (041), also prominent over (202), contribution was also observed, whereas the (220) peak showed no consistent monotonic trend (Figure S6).

Most changes in the fitted parameters occurred between approximately 300 and 900 µm from the external surface. This interval overlaps the expected transition from the outer fibrous region toward the HL; however, the current spatial registration does not support any specific assignment.

## 4. Discussion

The present observations show that the all-aragonitic shell of *C. gallina* is structurally heterogeneous across its thickness. The outer region comprises a convergent fibrous layer (CL), a divergent fibrous layer (DivL), and a porous transition region (DissL), whereas the inner region (HL) has a distinct nanogranular appearance and reveals a crossed-lamellar organization after etching. This inner architecture should not be described as nacre solely because it occupies a large fraction of the shell thickness: nacre requires diagnostic tablet-and-interlamellar morphology and typically exhibits a characteristic crystallographic texture.

Earlier studies of *C. gallina* distinguished outer and inner aragonitic microstructures and documented variation in porosity, fracture load, elastic response, strength, and fracture energy [33,35,36]. The present contribution extends that work by resolving the structure of the outer layer, its curved fibrous trajectories, subdivision boundaries, inverse-tulip units, and its association with fracture-surface morphology. Accordingly, the novelty lies in the detailed description of this hierarchical outer architecture rather than in the first mechanical or microstructural study of the species.

Based on structural evidence, we hypothesize that the outer layer may influence fracture propagation and associated energy dissipation while limiting potential mass loss due to fragmentation. This interpretation derives from post-fracture morphology of hammer-split specimens and does not establish the crack-initiation site, propagation sequence, dissipated energy, fracture toughness, or impact resistance. Several shell-scale mechanical properties of *Chamelea gallina*, including fracture load, elastic response, strength, and fracture energy, have already been reported in previous studies. Accordingly, the terms “cleavage plane” and “dissipation layer” are retained as working descriptors rather than demonstrated mechanical functions. The following section presents the crack-path hypotheses derived from the morphological observations.

### 4.1. Interpretation of Crack Propagation Pathways and Energy Dissipation

**Figure 10:**
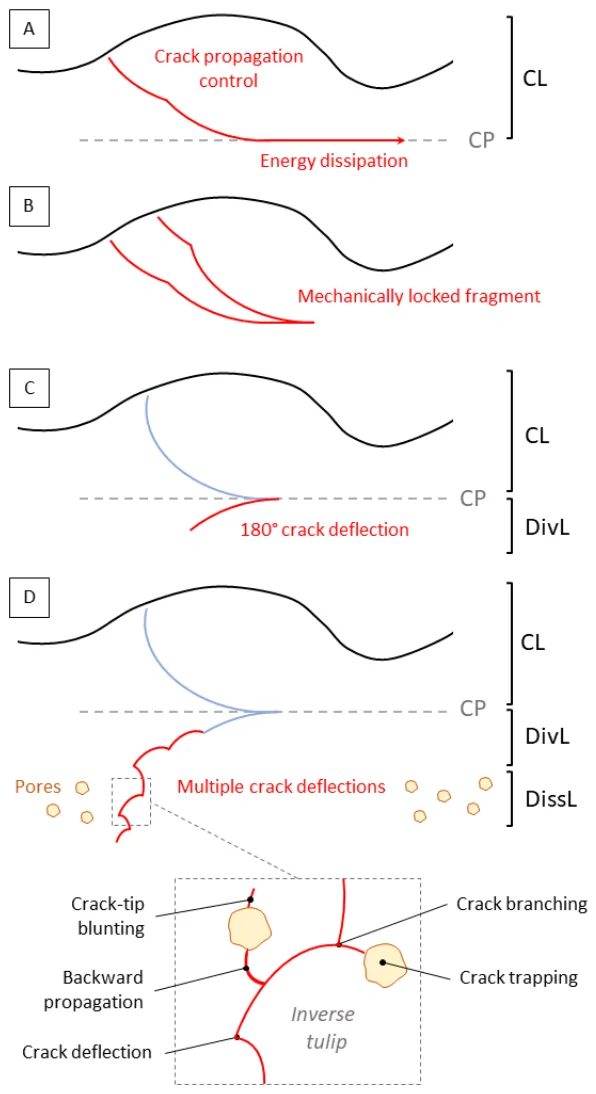
Conceptual hypothesis of fracture pathways associated with the hierarchical outer shell architecture: (**A**) crack propagation control in the CL and energy dissipation along the CP, (**B**) formation of mechanically locked fragments, (**C**) crack deflection moving from the CL to the DivL, (**D**) different energy dissipation mechanisms in the DissL. The schematic is derived from the SEM morphologies in Figure 2, Figure 3, Figure 4 and Figure 5 and the additional SEM images provided in revised Figure S1; it does not represent a directly measured crack-propagation sequence or quantified energy-dissipation mechanism. In each panel, red lines identify the mechanism presented.

The post-fracture morphologies observed in hammer-split specimens are consistent with preferential separation along hierarchical interfaces and with changes in fracture-surface direction at the CL–DivL boundary. Across the CL, fracture surfaces followed interfaces at multiple hierarchical levels, including boundaries between macroscopic subdivisions, adjacent fibers, and aligned rows of nanogranules. The same structure–path relationship recurs at each level: interfaces redirect the observed fracture surface rather than permitting a straight path through the mineralized units. Their differing orientations determine the local path, with curved fibers and subdivision boundaries converging toward the CL–DivL cleavage plane (CP), where a marked change in fracture-surface direction is observed (Figure 10A).

When the observed fracture surface reached the CP, it frequently extended along this interface and remained localized within a well-defined plane. Because fibers and nanogranules change orientation across the boundary, this geometry is consistent with crack deflection toward the distal shell region rather than direct penetration into the inner layers (Figure 10C). Although the present post-fracture observations do not allow the sequence or energetic cost of crack propagation to be quantified, they support the interpretation of the CP as an interface that may promote crack deflection and damage localization. Accordingly, its potential contribution to energy dissipation is discussed as a structure-based interpretation rather than as a directly quantified mechanical function. Similar interface-mediated deflection, microcracking, and crack-bridging mechanisms are established in nacreous and crossed-lamellar shells [12,13,24,25,26,27].

The irregular fracture-surface morphology observed within the CL produces curved fragments whose geometry may favor mutual interlocking and reduce their tendency toward complete detachment. These features are consistent with a potential role of the CL in limiting material loss by fragmentation, although this interpretation remains morphology-based (Figure 10B).

If a fracture surface extends beyond the CP into the DivL, it encounters a denser network of interfaces created by the shorter fibers. This architecture could increase path tortuosity and favor deflection or branching, thereby providing a structural basis for future quantitative evaluation (Figure 10D).

The preferred alignment of nanogranules and their gradual rotation near fiber boundaries provide a possible geometric basis for this behavior. A path following an inter-fiber interface may be redirected toward an adjacent fiber, whereas a path intersecting a fiber may be guided toward the neighboring interface (Figure 10D). Repetition of these direction changes would increase path complexity.

Within the DissL, the increased interfacial area, radial nanogranule alignment, and microporosity provide additional candidate crack-steering features. The orientation of nanogranules within each inverse-tulip unit could redirect an observed path toward unit interfaces. Pores may also promote crack-tip blunting or crack trapping, as reported for other porous ceramics (Figure 10D) [42,43]. Together, these features motivate the hypothesis that the DissL may delay inward fracture propagation.

Once a crack reaches the HL, however, no preferential propagation pathways are clearly identified, and fracture proceeds without evident structural guidance.

### 4.2. Crystallographic and Structural Layering

From a structural perspective, the FTIR and XRD profiles clearly reveal through-thickness heterogeneity.

From a materials-design perspective, the FTIR profiles delineate regions with different ν_2_/ν_4_ ratios. Once independently validated, variations in the elastic response of adjacent regions could generate an interfacial mismatch that promotes crack deflection, consistent with well-established toughening mechanisms in heterogeneous layered composites [12,13,15,21].

The XRD data further support the presence of structural rearrangements, as reflected by variations in preferred crystallographic orientation. The observed variations provide evidence of crystallographic differentiation across the shell and are consistent with the broader crystallographic variability reported for biogenic aragonite [24,44].

The gradual evolution of the crystallographic features from the outer surface toward the middle of the shell reveals a continuous texture gradient within the external region. This structural variation is expected to promote crack deflection and enhance damage tolerance.

The pronounced preferred orientation in the XRD pattern assigned to the HL is consistent with the crossed-lamellar structure revealed by etching beneath the apparently homogeneous nanogranular morphology. Most structural variations occur between the outer region and the middle of the shell. The apparent coherent-domain size is smaller in the outer region than in the inner region. Because these Scherrer estimates include unresolved peak overlap and instrumental broadening, they cannot be translated directly into compliance, stiffness, or energy dissipation. Changes in peak position were observed for the overlapping (102)/(200) and (041)/(202) reflections, whereas the (220) reflection showed no consistent shift.

## 5. Conclusions

The shell of *Chamelea gallina* displays a hierarchical, all-aragonitic architecture in which the outer region comprises convergent and divergent fibrous structures followed by inverse-tulip microstructures. Nanogranules occur throughout the shell; in the outer region they coalesce and align into fibrous units, whereas no preferred orientation is apparent in unetched fracture surfaces of the inner homogeneous layer. Etching nevertheless reveals a crossed-lamellar organization in the inner layer.

Fracture surfaces are consistent with candidate crack-steering mechanisms, including reorientation, branching and deflection along interfaces, as well as possible crack trapping or crack-tip blunting near pores. The frequently exposed plane between the two fibrous layers may localize the observed fracture path. The curved geometry of some fragments may also result in limited mass loss through mechanical interlocking. These interpretations are morphological hypotheses.

However, the present work contributes a detailed multiscale description of the outer shell architecture and a testable structure–fracture framework. It thereby provides a basis for investigating the evolution and biological role of this mineralized tissue and for translating graded orientation, hierarchical interfaces, and localized porosity into mechanically robust bioinspired materials.

## Data Availability

The original contributions presented in this study are included in the article/Supplementary Material. Further inquiries can be directed to the corresponding authors.

## Supplementary Materials

The following supporting information can be downloaded at: https://www.mdpi.com/article/10.3390/biom16091331/s1, Figure S1. Optical micrograph of the square-wave-like profiles and SEM images of the sagittal and transversal sections; Figure S2. SEM-EDS analysis of a polished shell section; Figure S3. EDS maps of Ca (orange) and Sr (green); Figure S4. Full set of XRD pattern collected along the transverse section: Figure S5. Full set of XRD pattern collected along the sagittal section; Figure S6. Position of the (220) reflection along the sagittal cross-section.
