# Supplementary material for: The Hierarchical Organization of the Layered Fibrous Shell of Chamelea gallina Guides Fracture Pathways
Source: Biomolecules. 2026 Sep 13;16(9):1331. doi: 10.3390/biom16091331 (PMC13604281; doi:10.3390/biom16091331)
Supplement: Supplementary file 1 [file biomolecules-16-01331-s001.zip › biomolecules-4505396-supplementary.pdf]

Supporting Information

The hierarchical organization of the fibrous shell layer of *Chamelea gallina* guides fracture pathways

*Devis Montroni *, Emilio Catelli, Silvia Prati, Arianna Mancuso, Stefano Goffredo, and Giuseppe Falini.*

Figure S1. Optical micrograph of the square-wave-like profiles and SEM images of the sagittal and transversal sections. P.2

Figure S2. SEM-EDS analysis of a polished shell section. P.3

Figure S3. EDS maps of Ca (orange) and Sr (green). P.4

Figure S4. Full set of XRD pattern collected along the transverse section P.5

Figure S5. Full set of XRD pattern collected along the sagittal section. P.6

Figure S6. Position of the (220) reflection along the sagittal cross-section. P.7

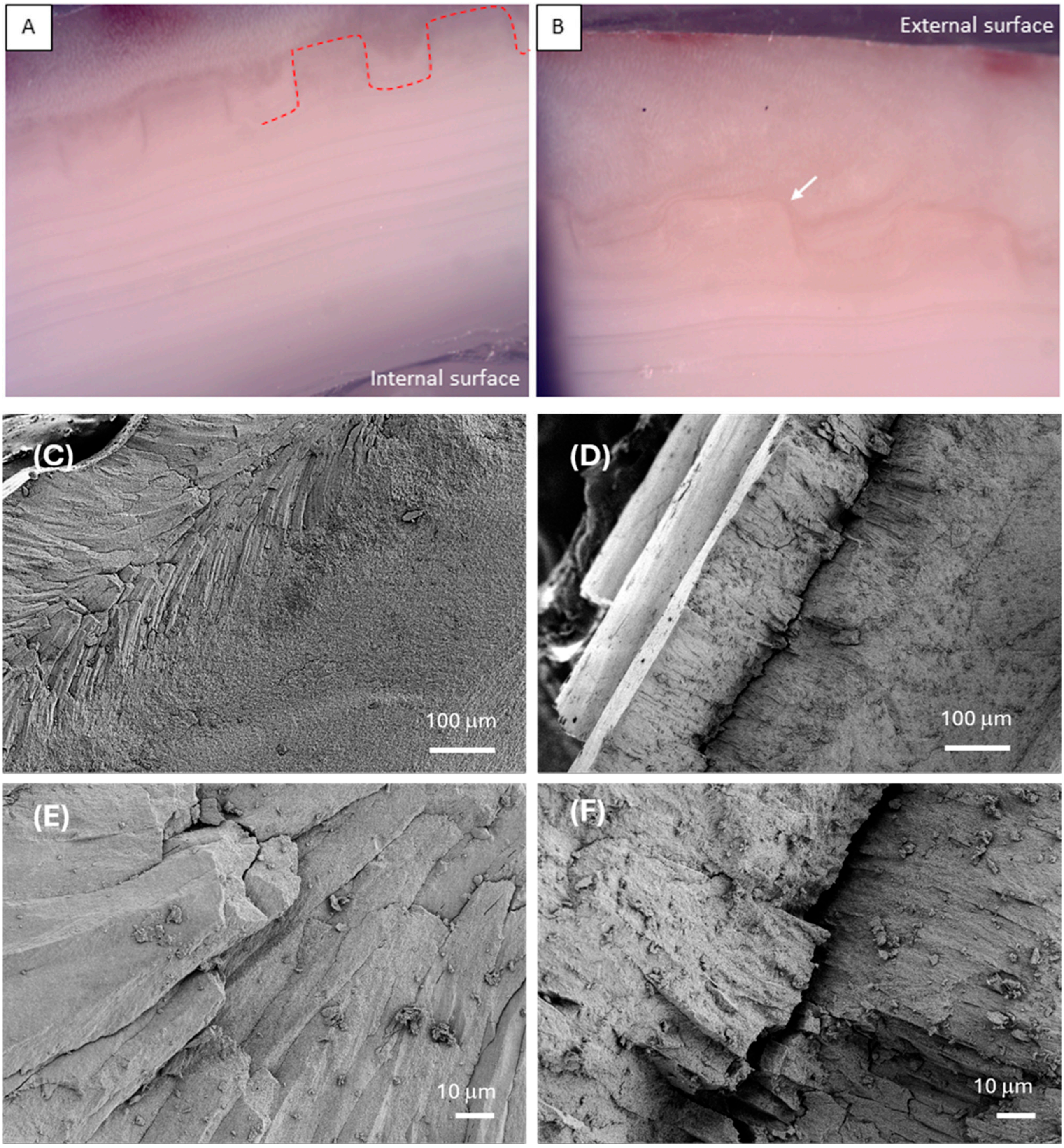

Figure S1. Optical micrograph (A and B) of a polished sagittal shell section showing the square-wave-like profiles. SEM image of the sagittal (C and E) and transversal (D and F).

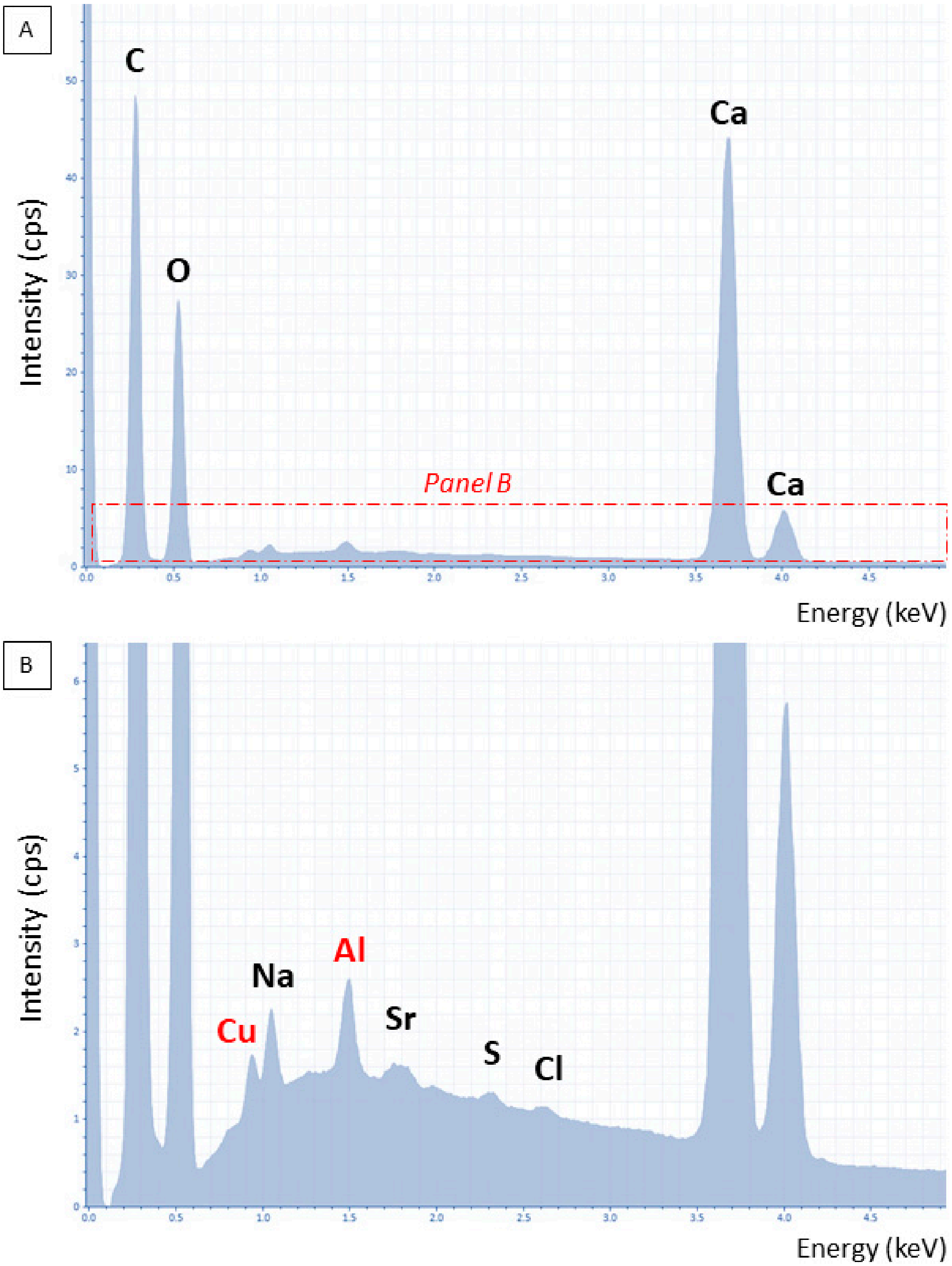

Figure S2. SEM-EDS analysis of a polished shell section. Signals shown in red were tentatively assigned to contamination from the alumina polishing suspension or the copper tape used on the SEM stub and were excluded from interpretation.

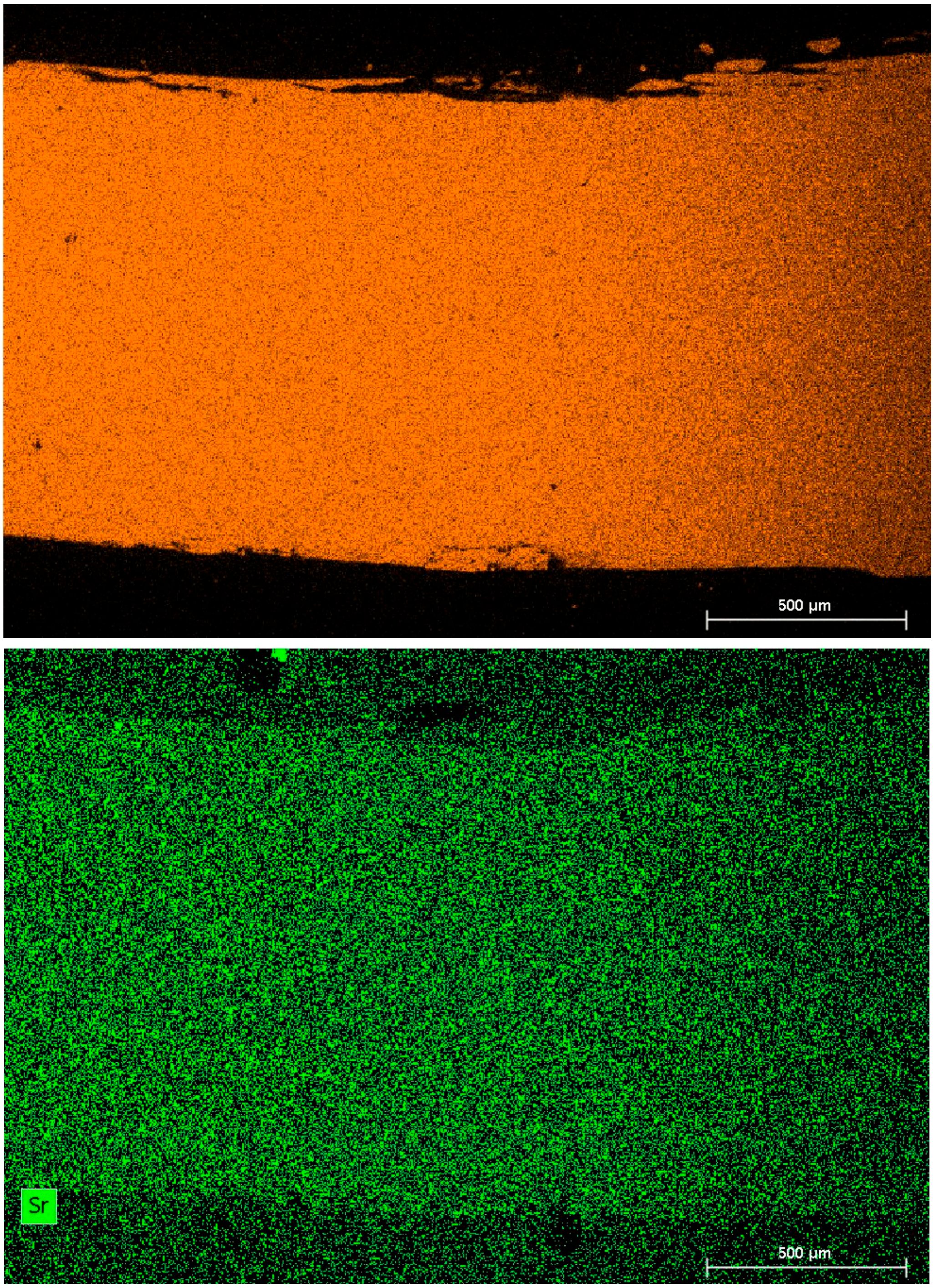

Figure S3. EDS maps of Ca (orange) and Sr (green) from a polished shell section; no systematic through-thickness intensity trend was resolved.

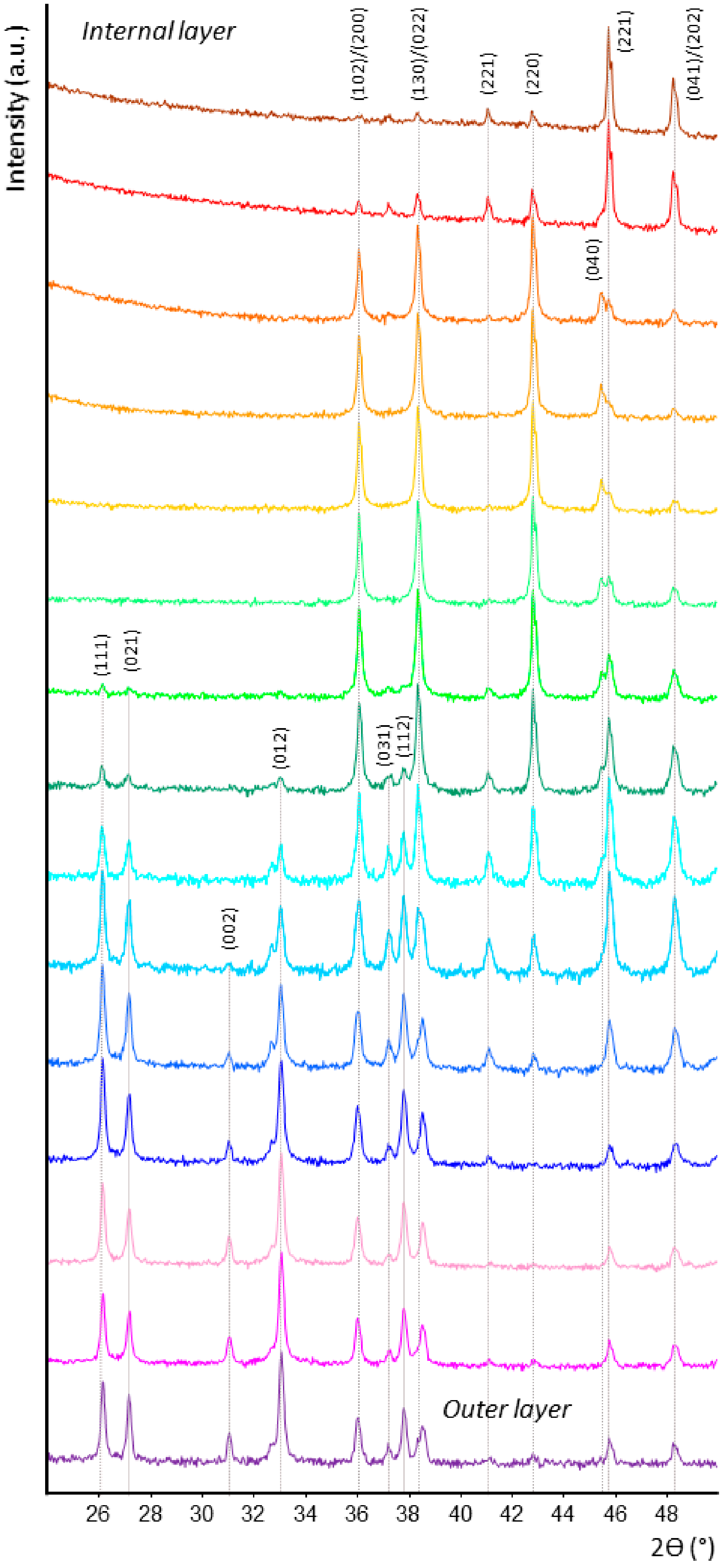

Figure S4. Full set of XRD pattern collected along the transverse section.

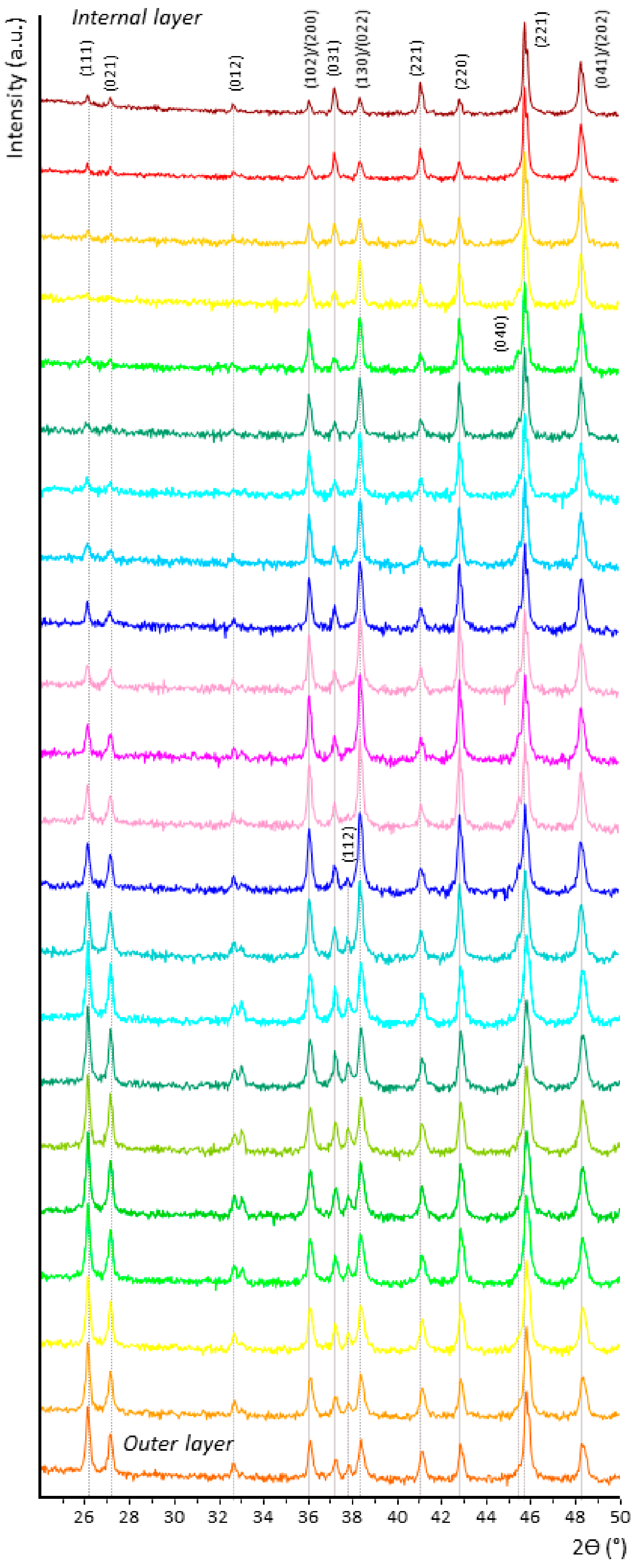

Figure S5. Full set of XRD pattern collected along the sagittal section.

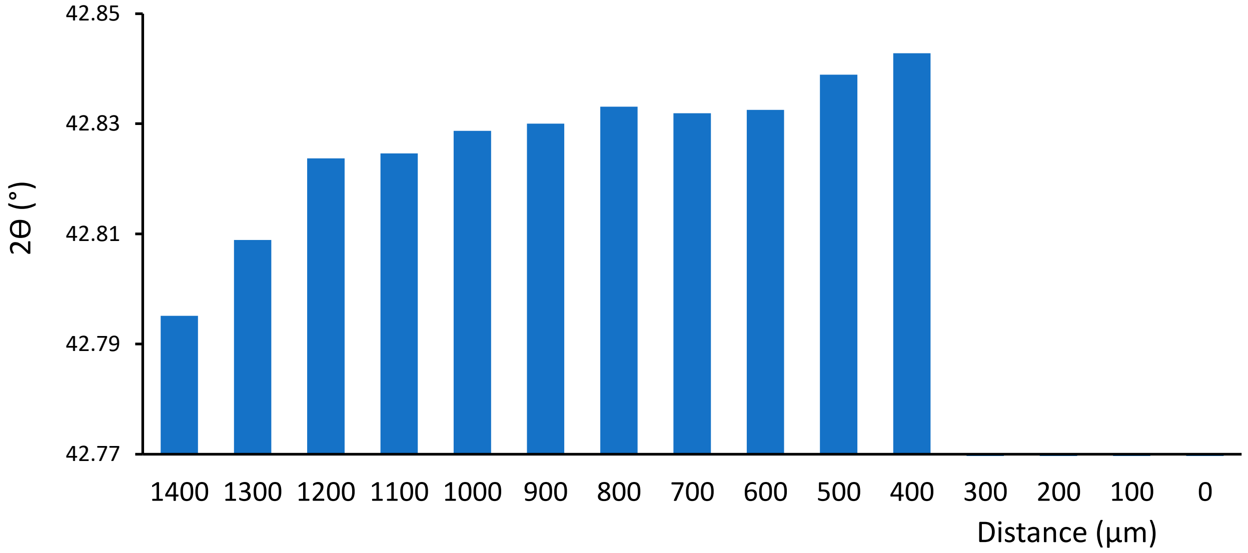

Figure S6. Position of the (220) reflection along the sagittal cross-section. Distance zero corresponds to the external surface. No (220) peak was resolved at distances <400 µm.
